# Party keepers: a significant community-based intervention for harm reduction

**DOI:** 10.1186/s13584-022-00535-8

**Published:** 2022-06-03

**Authors:** Roy Zucker, Zohar Mor, Anuar Abudin, Glen Davis, Hansel Arroyo, Gal Wagner Kolasko, Dan Arad, Guy Shilo

**Affiliations:** 1 Mount Sinai Hospital, Icahn School of Medicine New York, 1 Gustave L. Levy Pl, New York, NY 10029 USA; 2 Magen David Adom Israeli EMS Services, Tel-Aviv, Israel; 3 Tel Aviv Department of Health, Ministry of Health, Tel Aviv, Israel; 4 School of Health Sciences, Ashkelon Academic College, Ben Zvi 12, 78211 Ashkelon, Israel; 5 Private Practice, New York, USA; 6 Clalit Health Services, Zamenhof 42, Tel-Aviv, Israel; 7 Tel Aviv Medical Center Sourasky Medical Center, 6 Weizman St., 6423906 Tel-Aviv, Israel; 8 Bob Shapell School of Social Work, Tel Aviv University, P.O.B 39040, Ramat Aviv, Tel Aviv, Israel

**Keywords:** Drugs, Harm reduction, Men who have sex with men, Israel

## Abstract

**Background:**

Gay men use recreational drugs more often than heterosexuals—especially at social events. While partying at a venue, partygoers are at risk of drug overdosing, without access to an emergency help. This study evaluates a unique and novel intervention aimed at training men who have sex with men (MSM) and transgender individuals who frequent parties, to provide immediate assistance on-site to partygoers who have overdosed.

**Methods:**

The Party Keepers (PK) course is a unique 4-h training course that provides the participants with tools to identify, prevent, and treat common medical syndromes that are associated with excessive substance use. Participants were asked to complete a questionnaire on their sociodemographic attributes; their sexual risk behaviour; pre-exposure prophylaxis (PrEP) use and drug-use behaviour before and after the course; and the emergency situations they encountered in party venues after the course.

**Results:**

Of the 85 participants who completed the training and left valid contact information, 52 (62%) completed the questionnaires. Their average age was 37.0 years, most lived in Tel-Aviv, and were single. Participants reported that, after the course, they reduced their own use of recreational drugs (cocaine, amyl nitrates, alcohol), reduced their sexual risk behaviours, and significantly increased their use of PrEP. Of all the PKs, 63% (N = 32) indicated that they now provided first-aid and other assistance to partygoers at public venues, which enhanced their sense of community responsibility. In the multivariate analysis, a high level of confidence as a PK, and the knowledge gained in the course, predicted the incidence of subsequent assistance to partygoers in emergency situations.

**Conclusions:**

The PK initiative—a harm-reduction intervention led by peers, aimed at fighting drug overdosing at gay venues—was useful in reducing drug use and sexual risk behaviours among the course participants. Most course participants also responded to drug-related emergency situations at gay parties, as a result. This evaluation of community health intervention within a sexual minority community can help health policy makers design more community based interventions and allocate resources to include community participants in harm-reduction policies.

## Background

Recreational drugs (also known as ‘club drugs’) are chemical substances that are used for enjoyment or leisure purposes, rather than medical reasons. The use of recreational drugs is becoming a significant public health problem, due to risk of addiction and possible overdoses, or *polypharmacy*.

Men who have sex with men (MSM) are more likely than heterosexual men to use certain substances—in particular (see “Appendix 1”), recreational drugs and those that facilitate sexual pleasure, known as *chemsex*, or *Party ‘n Play* (PnP) [1]. The most common recreational substances associated with party venues and chemsex sessions include synthetic amphetamine (methcathinone, mephedrone, crystallised methamphetamine—also known as ‘crystal-meth’, or ‘Tina’), and anaesthetics such as ketamine or Gamma-hydroxybutyrate / Gamma butyrolactone (GHB/GBL—sometimes called ‘G’, or ‘Gina’). Other common recreational drugs include 3,4 methylenedioxymethamphetamine (MDMA/ ‘Ecstasy’), cocaine, and hallucinogenic drugs such as lysergic acid diethylamide (LSD) [2]. While the use of party drugs such as MDMA/Ecstasy used to be persistently common at circuit and electronic music events at nightclubs and warehouses, some studies suggest that the use of other club drugs—such as LSD, ketamine, powder cocaine, and GHB—has significantly increased between 2016 and 2019 [3], especially among MSM living with HIV [4, 5].

Several theories have been put forward to explain the relatively greater prevalence of recreational drug use among MSM. These include their relatively greater social acceptance of drugs than in the heterosexual community, and *minority stress*—namely, the high levels of stress faced by lesbians, gays, bisexuals and transgenders (LGBTs), who are often stigmatised because of their sexual orientation or gender identity, and consequently use drugs to cope with social stressors [6, 7].

According to the Center for Disease Control (CDC), overdose deaths involving cocaine and other psychostimulants with abuse potential increased by 42.4% in the United States between 2015 and 2016 [8]. Data on fatalities due to central nervous system (CNS) depressants such as GHB/GBL is hindered by the lack of hospital toxicologic testing or of standardisation in the investigation, recording, and certification of drug-related deaths [9].

Unintentional overdose, or polydrug use combined with intensified physical activities such as dancing at parties, can lead to severe complications—including CNS depression, dehydration, cardiac dysfunction, or even death [10–12]. Medically trained personnel in hospital settings can assess overdose situations through cardiac monitoring, pulse oximetry, urinalysis, and testing of electrolyte imbalance and underlying medical disorders. However, such measures are not accessible to a layperson at a party venue, without basic medical training [13].

To respond to the possible risks posed by the use of recreational drugs among MSM in Israel, a unique community-based harm-reduction intervention was instigated in Tel Aviv, Israel in 2018, dubbed ‘Party Keepers’ (PK). The concept of harm reduction involves a tailored change in individual practices, with a view to reducing risk behaviours, such as drug use. The PK training programme—which is four hours long—was designed by the LGBT Tel-Aviv Centre in collaboration with the Israel’s national emergency medical services (EMS) (‘Magen David Adom’). To our knowledge, this type of intervention is novel, and has not been yet published. The PK intervention was further adopted by the 'night life coalition', the main LGBT party organizers in New York City. The course covers basic knowledge of the impact of different drugs, and equips participants with basic skills to identify overdose situations, and to advise partygoers at gay venues about prevention or reduction of their drug-use habits. Specifically, it provides basic instruction on how to respond to mental distress; behavioural changes and aggressiveness; seizures and neurological complaints; hyperthermia; and dehydration. It also addresses situations related to hypoglycemia, vomiting, aspiration, and loss of consciousness due to the use of CNS depressants (“Appendix 2”). Course participants are recruited from the gay community, and the instructors are trained paramedics from the Israeli EMS services. With the completion of two PK training courses over two years, this study aimed to assess the impact of the training among course participants, and to evaluate the participants’ actual performances.

## Methods and materials

Two PK courses were held between 2018 and 2019, with participants recruited through advertising in social media. The course was offered to MSM and transgender party-goers, that were asked to register for the training free of charge. In order to evaluate the impact of the PK training course, questionnaires were distributed among participants who shared a valid contact information with the instructors at the end of the course, such as phone number or an electronic address. The messages were distributed between January and March 2021 by email or text messages to their smartphones. After signing informed consent, participants were asked to complete an anonymous online questionnaire about their demographic characteristics; drug-use behaviors before and after the course; their personal experience with eight common chemsex and party substances (marijuana, GHB, MDMA, ketamine, cocaine, crystal meth); their consumption of alcohol; their incidence of sex under the influence of drugs; use of pre-exposure prophylaxis (PrEP); and whether they had ever required emergency treatment due to excessive use of drugs. Participants were also asked to evaluate the effect that the course had had on them (on a Likert 5-point scale, ranging from *Not at all* = 1 to *Very much* = 5), based on eight measures of knowledge and social/community effects. An Exploratory Factor Analysis with Varimax rotation of the eight items revealed three factors, which explained 75.3% of the variance of the effects of the course (“Appendix 3”): *Knowledge gained at the course* (3 items); *Awareness of the threat of drug and alcohol use* (2 items); and *Community responsibility and confidence* (3 items). Scores for each factor were calculated as the mean of the items of that factor: the higher the score, the more significant the impact of the course on the participant’s knowledge, awareness of the threat inherent in drug and alcohol use, and community responsibility and confidence.

Participants were also asked how confident they were as a PK, and how satisfied they were with the course (both on a 5-point scale, ranging from *Not at all* = 1 to *Very much* = 5), and whether or not they had had occasion to help partygoers in distress (at parties or social venues) after the course. Finally, they were asked to describe in detail one common intervention they had performed as PK after the course (an open-ended question).

### Statistical analyses

The first step in evaluating the programme was to gauge its personal impact on the PKs—including changes in their own risk behaviours before and after the course, which were compared by means of McNemar’s Chi-square test for categorical variables. The second step was to assess how many PKs had actually assisted partygoers in medical distress at LGBT social venues, and to compare them with those who had not. To this end, independent variables were compared by means of the Chi-square or the Student’s *t*-test for categorical and continuous variables, respectively. Variables whose *p* < 5% in the univariate analyses and age were included in the multivariate analysis, to identify attributes associated with supporting partygoers after the course. The open-ended questions were analyzed using content analysis, to provide descriptive statistics of the participants’ common interventions as PKs, and their need for follow-up training. The study was approved by the Institutional Ethical Review Board of Ashkelon Academic College in Israel.

## Results

### Sample characteristics

Of the 130 participants who had enrolled in the two courses, 85 (65.4%) shared valid contact details, and were invited to take part in the study. Of these, 68 (80%) began completing the questionnaire. Sixteen of them failed to complete the entire questionnaire, so the final sample for analysis comprised 52 participants. Their average age was 37 years (SD = 5.4, range: 27–53). Most participants self-identified as cisgender males, and almost all self-identified as gay (Table 1). Over half were single, and most held an academic degree.

**Table 1:** Sample characteristics

Variable	N	%
Gender		
Cisgender male Transgender	49 3	94.2 5.8
Sexual orientation		
Gay Bisexual Heterosexual	47 3 2	90.4 5.8 3.8
Marital status		
Single Monogamous relationship Open relationship	30 5 17	57.7 9.6 32.7
Residence		
Central Region (including TLV) Elsewhere	50 2	96.2 3.8
Education		
High school BA MA PhD	7 28 14 3	13.5 53.8 26.9 5.8
Income^a^		
No income Below average Average Above average	2 10 16 24	3.8 19.2 30.8 46.2
PK^b^ course		
September, 2018 June, 2019	20 32	38.5 61.5

^a^Average monthly income in Israel is ~ €2600

^b^PK, Party Keepers

### The effects of PK course on participants

The outcomes of the course were compared between participants who finished the training in 2018 and those who finished it in 2019. No statistically significant differences were found between the groups, thus we therefore merged the results of both groups. Most participants (N = 34; 65.4%) professed being very satisfied with the course, and only 4 (7.7%) were unsatisfied. Almost all participants (N = 50; 96.2%) reported they would recommend to their friends to enrol in future courses. Course participants also reduced their personal risk behaviours as a result of the course—mainly by decreasing their use of cocaine, alcohol and chemsex practices, and a significant increase in PrEP use (Table 2). While 21 (40.4%) participants reported that prior to the course they had occasionally needed emergency help due to overuse of drugs, only 2 (3.8%) found themselves in such a situation after the course (p < 0.001).

**Table 2:** Difference in substance use and risk behaviours, before and after the PK course (N = 52)

	Before PK course		After PK course		
	N	%	N	%	*P*-value^a^
Drug use					
Grass	43	82.7	40	76.9	0.38
GHB	38	73.1	33	63.5	0.27
MDMA	45	86.5	40	76.9	0.18
Ketamine	33	63.5	32	61.5	1.00
Cocaine	35	67.3	27	51.9	0.02
Crystal Meth	5	9.6	5	9.6	1.00
Viagra (sidendenyl nitrate)	34	65.4	28	53.8	0.11
Alcohol use	37	71.2	27	51.9	0.01
Sex under the influence of drugs	36	69.2	22	42.3	0.001
Emergency state due to drug use	21	40.4	2	3.8	< 0.001
Used PrEP	27	51.9	33	63.5	< 0.001

^a^McNemar Chi-square test; PK, party keepers; GHB, Gamma butyrolactone; MDMA, methylenedioxymethamphetamine

### Party-Keepers’ community activity

The majority of the sample (N = 33; 63.5%) provided first aid or assisted partygoers in emergency situations at social venues after the course. No significant differences were found in terms of demographic characteristics between participants who helped partygoers in distress after the course, and those who did not (Table 3). However, those who did exhibited greater confidence levels as PKs, gained more knowledge at the course, and felt a greater sense of community responsibility and self-confidence than those who did not. In the multivariate analysis, a high degree of confidence as a PK, and the knowledge gained at the course, were associated with engagement in helping partygoers in distress (Table 4).

**Table 3:** Differences between participants who helped partygoers as part of their PK volunteering, and those who did not (N = 52)

	Helped (N = 33)		Did not help (N = 19)		
Variable	N	%	N	%	*P*-value^a^
Single	16	48.5	12	63.2	0.3
Resident in Central Region	32	97.0	18	94.7	0.7
Academic education	27	81.8	12	63.2	0.1
Above average income ^c^	18	54.5	6	31.6	0.1

	M	SD	M	SD	*P*-value^b^
Age	36.06	4.7	37.7	6.3	0.3
Level of confidence as PK	3.74	0.8	2.7	0.7	< 0.001
Level of satisfaction with the course	4.07	1.0	3.7	0.8	0.2
***Effects of PK course***					
Knowledge gained in course	4.0	0.8	3.1	0.9	0.001
Perceived threat of drug and alcohol	2.2	1.2	2.0	1.0	0.5
Community responsibility and confidence	3.1	0.9	2.5	0.7	0.01

^a^Chi square test

^b^Independent *t*-test

^c^Average monthly income in Israel is ~ €2600

**Table 4:** Logistic regression predicting incidence of helping other clubbers following the Party Keepers course (N = 52)

Predictor	B	SE	OR	95% CI	*p*-value
Age	− 0.1	0.1	0.9	0.8–1.1	0.3
Level of confidence as PK	1.6	0.6	5.1	1.4–17.3	0.01
Knowledge gained in course	1.0	0.4	5.0	1.1–5.9	0.02
Community responsibility and confidence	0.4	0.4	0.8	0.2–2.3	0.4

CI, Confidence Interval for Odds Ratio (OR)

On average, each participant had assisted 5 partygoers in distress (*SD* = 3.6, range 1–12). All participants reported having involved in calming down the situation, reassuring the partygoer in question, and taking control of the situation:

…First, I helped by listening to what the guy had to say, trying to comfort him, and calming the other friends that had gathered round (#43, aged 28)

I saw someone who was very confused and disoriented—I think he had taken too much K—so I suggested that he sit down, join my friends. I calmed him down, and stayed with him till he got better. (#25, aged 34)

**Fig. 1:**
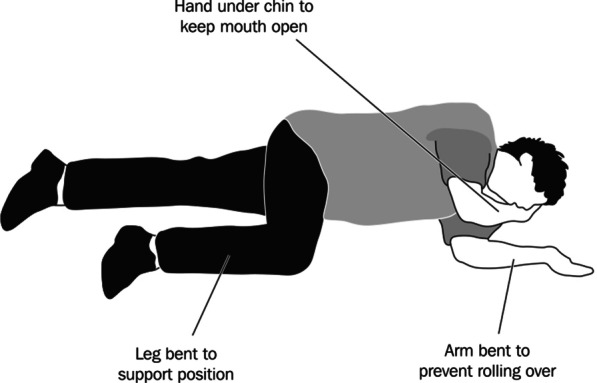
Recovery position

Eleven participants (50% of the participants who assisted partygoers after the course) reported having helped partygoers who were adversely affected by GHB—including loss of consciousness (known as ‘G-hole’). Specifically, they reported having cleared the space around the stricken clubber (sometimes helping him go outside), placing him in a ‘recovery position’ (Fig. 1), and keeping him seated erect, while he vomited:

At one party, someone got into G-hole—his friends thought they knew what to do, but I saw that they were in shock. I took over, cleared the crowd, placed the guy on a side sofa, and asked people not to touch him. I stayed to see that he wasn’t vomiting, and after a couple of minutes, he started talking. I woke him up and helped him sit down and recover. (#10, aged 33)

Someone fell down in the middle of the dance floor, after using G—his friends immediately disappeared. I moved everyone aside, leaned down to talk to him: he couldn’t talk and was very confused. With another friend of mine, we cleared the crowd that had gathered round, gave him some space and air, got him into a “recovery position”, and he got better after a while. We helped him onto his feet, went outside [with him] to the fresh air, talked with him a little, until I was sure he was better. (#26, aged 39)

Five participants (22.7%) reported dehydration as the most common state they encountered while intervening as PKs. In these situations, they reported that the partygoers they helped were disoriented and felt weak. They offered them something sweet to drink; two of them had electrolyte tablets on their person, and added one to the partygoer’s drink:

At one party, a guy was feeling unwell—he tried to drink, but I saw it wasn’t helping. I asked him how he was doing, and he said he was feeling weak and disoriented, although he had drunk a lot. I offered him some electrolyte tablets, added [one] to his water, and stayed with him while he was drinking and recovering. (#45, aged 47)

When I see people “drooping” at a party, I try to talk to them. If they react, I give them a lollypop (I bring a few to every party). In many cases, the sugar helps them, and they are “back to life” in seconds. (#15, aged 43)

In 2 cases of dehydration, participants called an ambulance, and stayed with the partygoer until it arrived.

Six participants (27.3%) stated that the most common situation they encountered was a mixture of drugs, or of drugs and alcohol. They reported having helped these clubbers by evacuating them from the club into the open air, and giving them sweet drinks. Two PKs described partygoers who lost consciousness after mixing several drugs, and helped by putting them in the recovery position, to reduce the risk of aspiration.

One participant reported having helped clubbers and friends on one occasion, by providing information and advice about the risks of combining drugs:

At a party, I saw a guy who mixed GHB with ketamine. He lost consciousness, and after several minutes started vomiting, and was at risk of choking. I told people around him to put him in a “recovery position”, and helped to clear his mouth. I stayed with him for at least half an hour, until he got better (#50, aged 37)

Of all the participants, 34 (65.4%) expressed interest in a follow-up course, which would provide further information about new substances in the gay scene, consolidate what they had learned from the previous course, and review the scenarios in which they had assisted partygoers.

## Discussion

Harm-reduction techniques are usually aimed at individuals with a view to reducing the risks and harms to themselves from substance use [14]. The PK initiative—involving training partygoers on addressing risk behaviours and common medical situations at gay party venues—is unique, inasmuch as it addresses the community aspects of the intervention. The initiative’s evaluation included an assessment of the changes in substance use and risky sexual behaviour among the participants in the PK courses.

In line with previous studies of harm-reduction strategies [14, 15], the results of this study found diminished use of cocaine, alcohol, and risky sexual behaviours (sex under the influence of drugs) among the course participants themselves, and a significant increase in their use of PrEP. In addition, it found a significant decrease in the number of emergency situations that the participants encountered at parties and social venues due to substance use. These outcomes are noteworthy, especially given the relatively meagre resources of the initiative, which consists of a 4-h course run by two professionals. At the individual level, the initiative may be defined both as a harm-reduction intervention, and as a prevention programme for a population at high risk of drug overdosing [14].

Unlike other harm-reduction initiatives—which target former substance users, or individuals diagnosed as having a drug-related problem—the PK initiative invited all partygoers in the LGBT community to take part, by encouraging them to enjoy the party, while being responsible for themselves and others. Thus, participants acted both as social agents who respond to drug overdosing on site, and as counsellors to their peers on how they may reduce the adverse impact of drugs. Although they reported high levels of satisfaction with the course, not all participants had the opportunity to identify or help out in emergency situations at parties or other LGBT-related venues after the course. Results from the multivariate analysis showed that knowledge and confidence gained at the course were associated with the participants’ subsequent intervention in emergency situations at gay venues. These findings illustrate the potential positive effect of harm-reduction initiatives that focus on education and understanding about the safe ways to use recreational drugs, instead of outright banning of their use, or other coercive interventions (psychological or psychosocial) [14].

Another benefit of the PK course is the self-empowerment that the participants reporting feeling as a result. In particular, they report that they took command and responded swiftly during emergency situations, while other partygoers stood by confused or embarrassed—or in some instances even abandoning their friend that was in distress. This sense of self-empowerment, along with that of community involvement, can shape the PKs’ gay identity, and define their social position as leaders.

While the course focused on emergency medical situations (such as hyponatremia, or hyperthermia), and basic tools to identify and treat those conditions, the participants’ reports mostly saw these as drug-related, and focused on external symptoms. For example, one PK described a person whom he claimed was disoriented from an overdose, when in fact the underlying medical condition may have been different. Laypersons who are not medically trained have limited capacity to accurately diagnose and treat emergency situations. Accordingly, future courses should include more practical, drug-oriented, and other differential diagnoses of common symptoms, and a lower threshold for calling for professional medical support.

The study has several limitations. First, the sample was relatively small, due to the limited number of participants who attended the first two courses, and only 52 of 130 who completed the PK training course took part in the study; thus, generalization to the wider gay population may be questionable. Second, study participants indicated their drug-use habits only following the course and not before, thus may be the cause of a report bias. Last, the cross-sectional nature of this study, and the fact that there was no control group, limits causality.

## Conclusions

In summary, the results of this study to evaluate PK course showed that this harm-reduction intervention was useful in reducing drug use and sexual risk behaviour among course participants, and prevented emergency situations related to drug use at parties and social venues from deteriorating any further. These results are encouraging, and support the notion of providing additional training, to expand PKs’ knowledge with periodic updates and data sharing. We also plan to increase the involvement of patrons to provide incentives for PKs—such as free entry, or drinks. With the gradual containment of COVID-19 pandemic and renewal of activities such as partygoing, this initiative may also be expanded to the other partygoers' population, not only in the gay community, such as Raves and Nature Parties. This evaluation of community health intervention within a sexual minority community can help health policy makers design more community based interventions and allocate resources to include community participants in harm-reduction policies.

## Data Availability

The datasets used and/or analysed during the current study are available from the corresponding author on reasonable request.

## Appendix 1

Main features of the three most common ‘club drugs’ among MSM.

**Table:**

	*MDMA/ECSTASY* (3,4 methylenedioxymethamphetamine)	*GHB/GBL* gamma-hydroxybutyrate/gammabutyrolactone	*KETAMIN*
Street name	MD, Molly, X, E, XTC	G, Gina, water, rape drug, soap, liquid ecstasy	K, Special K, KitKat, Vitamin K, Cat
Manner of use	Tablets or powder—mostly ingested/snorted	Transparent liquid (salty/bitter)	Snorted, or dissolved
T^1/2^	6–8 h	1–3 h	1–2 h
Onset of action	30–60 min	15–30 min	5–10 min
Action Mechanism	Serotonin, noradrenaline, dopaminergic	GABA and GHB-specific receptor	Antagonist of the N-methyl-D-aspartate (NMDA) receptor. Some opioid receptor activity
Favorable Clinical effects	Stimulant Empathy, energy, self-confidence, sensory awareness, inhibition of orgasm	CNS depressant euphoric, sexual enhancement, stimulant, relaxant effects	Dissociative drug Relaxation, dream-like state, visual distortion, dissociation
Toxic effects	Anxiety, dehydration, water intoxication, electrolyte imbalance, brain edema, rhabdomyolysis, disseminated intravascular coagulation (DIC), seizures, death	Agitation, vomiting, aspiration, sleepiness, respiratory depression, bradycardia, loss of consciousness (G-Hole), death	Imbalance, psychosis, respiratory depression, salivation, laryngospasm, apnea, bradycardia, death
Dependence	–	Physical mostly	–
Long term effects	Cognitive deficiencies, brain neurotoxicity	***Unknown***	***Unknown***

## Appendix 2

PK course main content.

**Table:**

Basic treatment to all emergency situations—check consciousness	
	1.Evacuate the patient to an open, cool space 2.Evaluate the person’s Airway, Breathing, and Circulation (ABC), maintain an open airway, and immobilise the person 3.If the person is not reacting to voice or physical stimulus—***call emergency services*** immediately, and search for an advanced level caregiver around you 4.Try raising both legs to a 45° angle for as long as the person is unconscious, for 1 min 5.Think and act upon preventable reasons for unconsciousness, 3Hs—**H**yperthermia, **H**ypoglycemia, and **H**ydration 6.If the person regains consciousness, monitor vital signs, mainly pulse and respirations, place the patient in recovery position (Fig. 1), and ask for transport to a nearby hospital

**Table:**

	Hypethermia	Syncope	Seizure	Drugs induced panic disorder	Hypoglycemia
Description	Uncontrolled increased body temperature that exceeds the body’s ability to lose heat	Rapid, brief loss of consciousness, due to acute global impairment in cerebral blood flow. Recovery is spontaneous	Paroxysmal event, due to abnormal excessive or synchronous neuronal activity in the brain	Emotional fear and anxiety that lasts for several minutes to an hour, due to drug use	A state in which blood sugar levels drop below normal values
Can be caused by the use of	MDMA, ecstasy, cocaine, methamphetamines + increased physical activity	MDMA, ecstasy, cocaine, methamphetamines	MDMA, ecstasy, cocaine, methamphetamines. Extreme hyponatremia, hyperthermia, or hypoglycemia can cause it	Cocaine, MDMA/ecstasy, amphetamines and hallucinogens	GHB/GBL, alcohol
External symptoms	Typical vital sign abnormalities (paleness, confusion, weakness)	Loss of consciousness	Physical, sensory or emotional changes (aura)	Chest pain, numbness, fear of dying, hyperventilation	Some altered level of consciousness: agitation or some level of non-responsiveness
Basic treatment	1.If available, place cold objects or ice on the person’s core 2.If conscious, hydrate with electrolyte-rich fluids 3.Monitor for potential neurologic change 4.Extreme situations may require transport to hospital	1.Treat in line with loss of consciousness 2.If possible, give something sweet 3.Upon full recovery—oral fluid 4.**Do not** splash liquid on face, or slap it	During seizure: 1.Remove harmful objects from surroundings 2.Do not remove the patient 3.Put something soft under their head 4.After recovery—use Recovery Position 5.Keep airway clear **After the seizure**: Calm patient, if fully conscious—look for a reversible cause. Give glucose. Consider treating Hypothermia or Hypoglycemia	1.Take the person to a quiet area 2.Calm the person down 3.Hydrate with glucose water 4.If not self-recovered for over an hour—call emergency services	1.If the person used GHB/GBL – hydrate with sucrose fluid 2.If consciousness is not regained – treat for hyperthermia, dehydration 3.If consciousness not regained – call emergency services

## Appendix 3

Factor loadings, communalities, and reliability—based on principal components analysis, with Varimax rotation for 8 items of the effects of the PK-course scale.

**Table:**

	Knowledge gained in the course	Awareness of the threat of drug and alcohol use	Community responsibility and confidence
The course:			
*Increased my knowledge of characteristics of party drugs*	0.90		
*Increased my knowledge of the effects of mixing drugs and alcohol*	0.85		
*Increased my knowledge of how to help people using drugs in emergency situations*	0.72		
*Increased my awareness of the threat of drugs*		0.88	
*Increased my awareness of the threat of alcohol*		0.86	
*Increased the number of my friends*			0.74
*Increased my self-confidence*			0.67
*Increased my sense of community commitment*			0.91
**Reliability (Cronbach α)**	**0.85**	**0.76**	**0.78**

Factor loadings < 0.2 are suppressed.

## Abbreviations

MSM: Men who have sex with men
PnP: Party 'n play
GHB/GBL/G: Gamma-hydroxybutyrate/Gamma butyrolactone
MDMA: Methylenedioxymethamphetamine
LSD: Lysergic acid diethylamide
LGBTs: Lesbians, gays, bisexuals and transgenders
CNS: Central nervous system
PK: Party Keepers
PrEP: Pre-exposure prophylaxis
